# Limb apraxia in Parkinson's disease and atypical parkinsonian syndromes: a systematic review

**DOI:** 10.1007/s10072-026-09215-w

**Published:** 2026-07-04

**Authors:** Evangelia Smaragdaki, Ioannis Stamelos, Magda Tsolaki, Nicolaos Scarmeas, Constantin Potagas, Leonidas Stefanis, Sokratis G. Papageorgiou, Vasilios C. Constantinides

**Affiliations:** 1 First Department of Neurology, Medical School, National and Kapodistrian University of Athens, Eginition Hospital, 11528 Athens, Greece; 2 Neurochemistry and Biomarkers Unit, First Department of Neurology, Eginition Hospital, National and Kapodistrian University of Athens, 11528 Athens, Greece; 3 Laboratory of Neurodegenerative Diseases, Center for Interdisciplinary Research and Innovation (CIRI), Aristotle University of Thessaloniki (AUTh), 54124 Thessaloniki, Greece; 4 Greek Association of Alzheimer’s Disease and Related Disorders (GAADRD), 54643 Thessaloniki, Greece; 5 Department of Neurology, Columbia University, New York, NY USA

**Keywords:** Apraxia, Parkinson’s disease, Corticobasal syndrome, Progressive supranuclear palsy, Multiple system atrophy, Atypical Parkinsonism

## Abstract

**Background:**

Evidence regarding the clinical manifestations, disease-specific profiles and diagnostic significance of limb apraxia in Parkinson's disease and Atypical Parkinsonian Syndromes (APS) is limited.

**Objectives:**

The present systematic review aims to consolidate current knowledge on limb apraxia across neurodegenerative disorders, including Parkinson’s disease (PD), corticobasal syndrome (CBS), Progressive Supranuclear Palsy (PSP) and Multiple System Atrophy (MSA).

**Methods:**

A systematic literature review was conducted in accordance with PRISMA guidelines. Studies were included if they enrolled ≥ 10 patients in at least one of the patient groups and ≥ 10 control subjects with quantitative data on apraxic deficits. Risk of bias assessment was assessed.

**Results:**

Twenty-two studies met inclusion criteria (PD *n* = 11; CBS *n* = 10; PSP *n* = 7; MSA *n* = 3). Across PD and APS, praxis assessment primarily involved gesture imitation, pantomime, action sequencing, actual tool use and measures of fine motor coordination. CBS demonstrated the most severe/widespread apraxic impairment, affecting both meaningless and meaningful (transitive and intransitive) gestures, as well as action sequencing and fine motor control. In PD, apraxic deficits were generally milder but shared overlapping features with CBS. Direct comparative studies between PD and CBS remain scarce. PSP was characterized by less frequent and predominantly sequence-related impairments, whereas findings in MSA were heterogeneous and less pronounced.

**Conclusions:**

Limb apraxia phenotypes differ across PD, CBS, PSP and MSA and may contribute to their differential diagnosis. Future research should adopt standardized, multimodal praxis assessment protocols in larger cohorts including all major neurodegenerative parkinsonian disorders, to facilitate the direct comparison of limb apraxia across these diseases.

## Introduction

Apraxia is the inability to perform specific and predefined actions or to carry out learned and purposeful movements, independently of sensory, motor and cognitive deficits that could impair the comprehension of the task, the recognition of the stimulus and the implementation of the response. Apraxia is due to acquired brain lesions and emerges as a discrepancy in accuracy between the intended action and the actual performance. Apraxia appears in daily activities and in standardized tests requiring actions to be performed on command and/or on imitation. Apraxia is not a unitary disorder with a unique neuropsychological basis. In clinical settings, different forms of apraxia exist. They share the label but reflect independent cognitive and neural mechanisms. Apraxia disorders can be specific to body parts (limb apraxia, oral apraxia, trunk apraxia), tasks (constructional apraxia, dressing apraxia) or functions (apraxic agraphia, apraxia of speech) [1].

Limb apraxia is a heterogeneous syndrome characterised by an impaired ability to execute learned movements, such as gestures and movements needed for skilled use of tools. Traditionally, limb apraxia has been subdivided into ideational apraxia (IA) and conceptual apraxia (CA), which mainly reflect conceptual deficits, as well as ideomotor apraxia (IMA) and limb-kinetic apraxia (LKA), which predominantly reflect deficits in movement production and execution.

Clinical manifestations of different types of apraxia may present as follows [2]:

- IA: impaired ability to identify and execute the correct sequence of actions necessary to accomplish a task or to use a tool, even when provided with a list of required steps.
- CA: impaired ability to identify the appropriate tool for a given task, pantomime the correct utilization of a tool and impaired recognition of its purpose. (In some models, CA is considered closely related to IA).
- IMA: impaired ability to execute gestures (on imitation and on verbal commands) and pantomimes of tool use, deficits in spatiotemporal orientation and positioning. Movements involving their fingers, hands, and arms display abnormal trajectories.
- LKA: impaired ability to execute the fine, coordinated movements needed to manipulate a tool, resulting in impaired dexterity and clumsy movements.

It should be noted that alternative definitions and classifications of ideational, conceptual, ideomotor and limb-kinetic apraxia have been proposed, and no universally accepted taxonomy currently exists. For the purposes of the present review, we adopted the above classification because it is commonly used in studies investigating praxis dysfunction in parkinsonian disorders.

Oral apraxia may be defined as the inability to perform voluntary movements with the muscles of the larynx, pharynx, tongue, lips and cheeks, although automatic movements of the same muscles are preserved [3]. Trunk movement impairments, labelled ‘trunk apraxia’, have been reported within a syndrome associated with (bilateral) frontal lobe lesions which encompassed stance and gait apraxia [4]. Constructional Apraxia indicates the inability to accurately reproduce two-dimensional or three-dimensional visual models [5]. Dressing apraxia is defined as the condition in which automatic, spontaneous capacity for daily dressing is lost and the body relationships of the upper and lower directions, two sides, or left and right orientation of clothes become disordered, making the task of dressing impossible [6]. Pure agraphia represents a rare neuropsychological syndrome in which writing is selectively impaired while other language modalities remain intact, providing a unique window into the specialized cognitive and neural mechanisms that support written language production [7]. Apraxia of speech is a motor speech disorder that reflects disruptions in motor planning and programming [8].

Although several forms of apraxia have been described, the present review primarily focuses on limb apraxia and related praxis disturbances in parkinsonian disorders. Other syndromes, such as constructional, dressing, trunk and oral apraxia, pure agraphia and apraxia of speech are discussed only when directly relevant to the studied included. For reasons of brevity, the term apraxia will be used in reference to limb apraxia throughout the manuscript, unless otherwise specified.

Anatomically, apraxia is widely conceptualized as a fronto-temporoparietal network disorder of the left hemisphere [9]. However, due to the bilateral representation of learned motor actions, symptoms may be observed on both sides of the body [10]. Nevertheless, knowledge of the pathophysiological mechanisms underlying the different manifestations of apraxia has been derived almost exclusively from studies in patients with ischemic stroke (abrupt, focal, clearly demarcated, destructive lesion), most commonly of left middle cerebral artery distribution [10]. Studies investigating the manifestations and anatomical substrates of apraxic deficits in different neurodegenerative diseases (progressive, diffuse, not clearly defined atrophy), as well as possible differences among these diseases, are scarce. Given the diverse patterns of cortical and subcortical degeneration observed in neurodegenerative movement disorders such as Parkinson’s disease and Atypical Parkinsonian Syndromes (APS) including corticobasal syndrome, Progressive Supranuclear Palsy and Multiple System Atrophy, differences in both clinical manifestations and the prevalence of apraxic deficits are to be expected. Furthermore, patients with heterogeneous diseases are expected to exhibit both qualitative and quantitative differences in performance on different tests that assess apraxia.

Parkinson’s disease (PD) is a neurodegenerative movement disorder that is increasing in prevalence especially as the population continues to age [11]. Patients with PD primarily present motor symptoms such as bradykinesia, rigidity, and tremor, due to the progressive degeneration of dopaminergic neurons in the substantia nigra [12]. Although apraxia is considered a red flag for PD, evidence suggests that upper limb apraxia may emerge, particularly in the later stages of the disease or in patients with concurrent cognitive decline [13].

Existing data regarding apraxia in PD are conflicting, and there is a risk of overestimating the prevalence or severity of apraxic deficits due to the confounding effects of primary motor symptoms and the methodological limitations of current assessment tools used in clinical settings. The development and implementation of refined assessment protocols, capable of dissociating apraxia from pure motor disturbances, are essential.

Corticobasal Degeneration (CBD), a 4-repeat (4R) tauopathy [14], which involves the dysfunction of both cortical and subcortical structures, manifests with motor symptoms and cognitive dysfunction. MRI often reveals asymmetric cortical atrophy, most prominently affecting the frontoparietal regions [15].

Clinically, CBD manifests most frequently as corticobasal syndrome (CBS), though it may also present as other phenotypes, (e.g. a frontal behavioral-spatial syndrome) and progressive non-fluent aphasia (PNFA), among others) [16]. Importantly, CBS is a clinical syndrome which may have diverse underlying pathologies including Alzheimer’s Disease (AD), progressive supranuclear palsy, other tauopathies and TDP-43 pathology. Distinguishing CBS due to AD versus non-AD pathology based solely on apraxia and oculomotor abnormalities is challenging [17].

Limb apraxia is considered a core clinical feature of CBS and is often among the earliest signs and contributes significantly to functional impairment. Epidemiologically, apraxia is present in approximately 70–80% of CBS cases [18]. Clinically, it often manifests as a unilateral inability to perform pantomimes, either to verbal command or on imitation. However, bilateral apraxia may also be evident, even in the early stages of disease, particularly when degeneration is more diffuse.

Progressive supranuclear palsy (PSP) is another 4R tauopathy. The most common phenotype, Richardson’s syndrome, presents with early postural instability and oculomotor abnormalities [19]. PSP, however, is now recognized as a clinically heterogeneous disease, with several distinct phenotypes, including Progressive Supranuclear Palsy-Corticobasal Syndrome (PSP-CBS), which closely mimics corticobasal syndrome in both clinical presentation and anatomical distribution of neurodegeneration [20].

Patients with PSP-CBS may fulfill criteria for both CBS and Richardson syndrome. This clinical overlap reflects the involvement of frontoparietal networks in both PSP and CBS and highlights the limitations of attributing apraxia solely to parietal lobe dysfunction or CBD pathology. This phenotypic convergence between CBS due to CBD and PSP-CBS further complicates clinical diagnosis and underscores the need for disease-specific biomarkers [20].

Multiple System Atrophy (MSA) is a sporadic neurodegenerative disorder characterized by varying degrees of parkinsonism, cerebellar ataxia, and autonomic dysfunction. [21]. Apraxia is not a prominent or core feature of MSA, although some patients may exhibit task-related motor planning difficulties, particularly in advanced stages, which may resemble apraxic deficits. Assessment of apraxia is particularly difficult, considering the presence of cerebellar dysfunction, bradykinesia, and executive dysfunction in MSA [22]. Studies investigating the manifestations of apraxia in MSA are limited.

Distinct phenotypes of apraxia can be observed in patients with PD and APS. While apraxia is a well-established core clinical feature of CBS [16], its prevalence and specific manifestations in PD, PSP and MSA remain less clearly defined and are often inconsistently reported across studies.

### The present study

The present systematic literature review aims to consolidate and critically appraise current evidence regarding apraxia in PD, CBS, PSP and MSA. The purpose of the present study is primarily to investigate clinical manifestations reflecting different types of apraxic deficits and secondarily diagnostic significance of apraxia across these disorders. Given the phenotypic variability of neurodegenerative movement disorders, such as the presence of CBS phenotypes in both CBD and PSP, accurate diagnosis of apraxia may offer valuable diagnostic insights and help differentiate between overlapping clinical presentations.

Moreover, this review highlights the challenges posed by the lack of standardized, disease-specific assessment tools for apraxia, especially in PD, PSP and MSA, where motor or cognitive symptoms may obscure or mimic apraxic deficits. By synthesizing findings across diverse studies, the review seeks to uncover patterns that may refine diagnostic criteria and promote the development of structured screening and rehabilitation protocols. Ultimately, the goal is to underscore the clinical relevance of apraxia not only as a marker of disease burden but also as a potential tool for improving differential diagnosis, monitoring disease progression and enhancing patient-centered therapeutic strategies.

## Materials and methods

The PRISMA statement (Preferred Reporting Items for Systematic Reviews and Meta-Analyses) was followed for this Systematic Review [23]. No institutional board review approval was obtained, since this study did not utilize novel data and was based exclusively on previously published data.

### Literature search strategy

PubMed and Web of Science were searched from database inception by three researchers independently (E.S, I.S. and V.C.C). A consensus among reviewers regarding study eligibility was required for study inclusion. In cases of full-text unavailability, corresponding authors of papers were contacted.

The search strategy applied was as follows:

((corticobasal) OR (extrapyramidal) OR (supranuclear) OR (Richardson) OR (Parkinson) OR (multiple system atrophy) OR (parkinsoni*)) AND (apraxia) in the study title or abstract.

### Eligibility criteria and study selection

The inclusion criteria were as follows:

- Publication in English language;
- Original, peer-reviewed research papers;
- Studies with ≥ 10 subjects in at least one of the patient groups (PD); (PSP); (CBS); (MSA) and ≥ 10 control subjects (ctrl).

The exclusion criteria were as follows:

- Non-original studies (reviews, meta-analyses);
- Case reports;
- Abstracts at conferences, congresses of other scientific meetings;
- Studies with identical patient cohorts and similar results.

In cases of publications with complete or partial overlap of patient cohorts, an algorithm was applied to reach a decision regarding the eligibility of the studies [24]. This algorithm included an evaluation of authorship, study characteristics, sample characteristics, constructs’ and measures’ definitions, and study effects.

After this initial search, a search was performed manually on all included studies, which included (a) citations of included studies; (b) references of included studies; (c) most relevant studies (from PubMed); (d) studies included in systematic reviews and meta-analyses performed in the past.

### Data extraction

Data extraction was performed by two authors independently (E.S. and I.S.). All data were cross-checked for discrepancies among databases, and raw data from the manuscripts were re-evaluated when necessary.

The following data were extracted from the studies: first author; year of publication; methods of assessing different types of apraxic deficits; disease groups (*n*); control group (*n*); main findings regarding apraxia in PD, CBS, PSP and MSA and control subjects, apraxic deficits and diagnostic significance of apraxia in PD and APS.

### Risk of bias assessment

The methodological quality of the included studies was assessed using the Newcastle–Ottawa Scale (NOS) for observational studies. This tool evaluates study quality across three domains: a) selection of study groups (maximum four stars); b) comparability of groups (maximum two stars) and c) ascertainment of exposure/outcome (maximum three stars). The maximum possible score is nine stars. Studies scoring 7–9 stars were considered high quality, 5–6 moderate quality and ≤ 4 low quality.

## Results

### Literature search and screening results

**Fig. 1:**
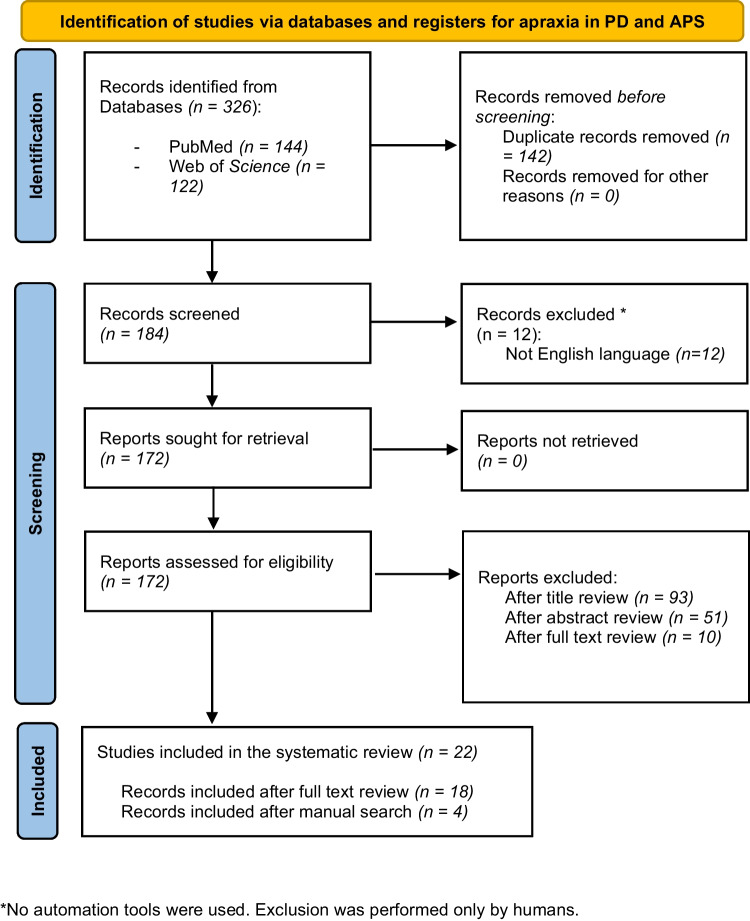
Flow chart of study selection according to PRISMA (Preferred Reporting Items for Systematic Reviews and Meta-Analyses) criteria

A total of 326 papers were retrieved from the PubMed and Web of Science. After the elimination of duplicate records, 184 records were screened by a review of the title and abstract. Twelve studies were eliminated due to being written in a non-English language, ninety-three after the title review and fifty-one after the abstract review. A total of twenty-eight studies were reviewed based on their full texts, resulting in the exclusion of ten further studies. In total, twenty-two studies were included in the systematic review (eighteen after a full-text review and four after a manual search) (Fig. 1).

### Characteristics of included studies

The basic characteristics of the included studies are summarized in Table 1. Twenty-two studies were included in the systematic review (Table 1).

**Table 1:** Characteristics of studies included in the systematic review

Study number	Study	Praxis assessment method(s)	Disease groups/controls *(n)*	Main findings
1	Sharpe, 1983	Gestural Test based on the work of Kaplan	15 PD;15 ctrls	Level of gestural representation score: PD < ctrl [*F* (1, 28) = 7.27, *p* < 0.05]; Spatial errors: PD > ctrl [*F* (1, 28) = 14.02, *p* < 0.001]
2	Goldenberg, 1986	Imitation of gestures; Pantomime of symbolic gestures and object use	42 PD; 38 ctrls	IMA: PD > ctrl (*p* < 0.0003)
3	Grossman, 1991	Boston praxis battery	22 PD; 11 ctrls	Performance: PD < ctrl on measures of representational praxis (U = 360. 0; *p* < 0.005) and nonrepresentational praxis (U = 364.0; *p* < 0.01)
4	Leiguarda, 1997	Imitation and pantomime of meaningful and meaningless gestures; Movement imitation test (MIT); Multiple step tasks	45 PD; 12 PSP; 10 MSA; 12 DIP; 50 ctrls	Bilateral IMA for transitive movements: 27% of PD patients (*p* = 0.0001); MIT in PD with < without apraxia (*p* < 0.0001); 8 of 12 PSP patients (*p* < 0.006) using a cut-off score of 26
5	Monza, 1998	De Renzi ideomotor apraxia test	19 MSA; 19 PSP; 14 PD; 18 ctrls	IMA: ctrl < PD < MSA < PSP (*p* < 0.0001)
6	Pharr, 2001	Test of Oral and Limb Apraxia	14 PSP; 13 CBD; 12 ctrls	Apraxic errors: PSP&CBD > ctrl (*p* < 0.001 in both cases); Severity: CBD > PSP (*p* < 0.001)
7	Peigneux, 2001	Shortened version of the “Batterie d’Evaluation des Praxies”	18 CBD; 15 ctrls	Global mean performance: CBD < ctrl (*p* < 0.001) Correction scores regarding error types irrespective of the condition: CBD < ctrl (*ps* < 0.005)
8	Soliveri, 2003	De Renzi ideomotor apraxia test	24 CBD; 25 PSP; 19 ctrls	Total apraxia score: CBD < PSP&ctrl (*p* < 0.0001). Distal < proximal gesture scores: CBD patients (*p* = 0.007)
9	Soliveri, 2005	De Renzi ideomotor apraxia test	24 CBD; 25 PSP; 19 ctrls	IMA scores with most compromised arm: CBD < PSP < ctrl (*p* < 0.001) IMA scores with least compromised arm: CBD = PSP < ctrl (*p* < 0.001)
10	Soliveri, 2005	De Renzi ideomotor apraxia test	25 PSP; 11 D-PSP; 14 ND-PSP; 19 ctrls	Total and partial apraxia Scores: D-PSP < ND-PSP < ctrl (*p* < 0.001) ND-PSP patients: significant > nonsignificant gestures (*p* = 0.003)
11	Boxer, 2006	Clinical examination; MMSE	14 CBD; 15 PSP; 80 ctrls	limb apraxia: CBD > PSP (*p* < 0.001)
12	Borroni, 2008	De Renzi ideomotor apraxia test	20 CBS; 21 ctrls	Affected limb at onset: R > L (55% > 45%) Bilateral > right unilateral despite an asymmetrical onset. De Renzi test: Mean (SD) test scores = 51.0 (23.8) for the right limb and 52.6 (21.9) for the left limb
13	Gebhardt, 2008	Coin rotation (CR) & finger tapping (FT) tasks	12 PD; 12 ctrls	Dopaminergic treatment: FT scores > CR scores (up to the level of controls) (F_1,22_ = 7.9, *p* = 0.01; η^2^ = 0.26) CR scores both in OFF and ON: PD < ctrl (*p* < 0.001)
14	Huey, 2009	Test of Oral and Limb Apraxia (TOLA)	48 CBS; 14 ctrls	TOLA: CBS < ctrl (TOLA imitation: *t* = 13.6, *p* = 0.01; command: *t* = 11.4, *P* = 0.01; oral: *t* = 6.1, *p* = 0.01; limb: *t* = 13.6, *p* = 0.01)
15	Foki, 2010	Coin rotation (CR) task	10 PD; 10 ctrls	Frequency: ctrl > PD (t = 6.349, *p* < 0.001)
16	Uluduz, 2010	The Mayo Clinic praxis test; Luria test; use of actual objects	19 PD; 16 MSA; 25 ctrls	Mean total praxis score: MSA (75.9 ± 18) < PD (92.4 ± 4) < ctrl (97.4 ± 2) (*p* = 0.000). Transitive performances of upper extremities and sequential Tasks: PD < ctrl (*p* < 0.05). Subgroup praxis scores: substantially worse in MSA group (*p* < 0.0001)
17	Stamenova, 2011	Conceptual and Gesture production limb apraxia assessment	10 CBS-rhp; 7 CBS-lhp; 28 ctrls	Significant difference among the means of the three groups on all gesture production tasks, but Pantomime of Intransitive Gestures (RHP = LHP < ctrl, *p* < 0.005)
18	Qureshi, 2011	Action sequencing picture tasks	10 PD; 10 ctrls	Action-sequencing tasks: PD < ctrl (*p* < 0.05)
19	Burrell, 2014	Imitation of meaningful and meaningless hand gestures	17 CBS; 17 ctrls	Production of meaningful and meaningless hand gestures: CBS < ctrl
20	Kübel, 2017	Coin rotation (CR) task	22 PD; 13 ctrls	PD < ctrl (*p* < 0.001)
21	Vanbellingen, 2018	Coin rotation (CR) task	80 non-demented PD patients	PD < ctrl (*p* < 0.0001)
22	Jo, 2021	Adaptation from the Florida Apraxia Battery	52 CBS; 13 ctrls	Apraxia subtype: both ideomotor and imitation apraxia (DM group, n = 22, 42.3%) > ideomotor apraxia only (D group, n = 15, 28.8%) = imitation apraxia only (M group, n = 15, 28.8%). Probable CBS and Probable PSP: DM > D = M, 45.5% vs. 13.3% vs. 13.3%, *p* = 0.036; 59.1% vs. 13.3% vs. 13.3%, *p* = 0.016)

*PD* Parkinson’s disease; *ctrls* Control participants; *ctrl* Control group; *IMA* Ideomotor apraxia; *MIT* Movement imitation test; *DIP* Drug induced parkinsonism; *PSP* Progressive supranuclear palsy; *MSA* Multiple system atrophy; *CBD* Corticobasal degeneration; *D-PSP* Demented PSP patients; *ND-PSP* Non-demented PSP patients; *MMSE* Mini mental state examination; *CR* Coin rotation task; *FT* Finger tapping task; *TOLA* Test of oral and limb apraxia; *RHP* Right hemisphere predominance; *LHP* Left hemisphere predominance; *CBS* Corticobasal syndrome; *D group* Patients with ideomotor apraxia only; *M group* Patients with imitation apraxia only; *DM group* Patients with both ideomotor and imitation apraxia

The methodological quality of the included studies ranged from moderate to high according to the Newcastle–Ottawa Scale, with scores ranging from 6 to 9. Most studies demonstrated adequate case definition and appropriate selection of control groups, although comparability between groups was sometimes limited due to insufficient adjustment for potential confounders such as disease severity or cognitive status (Table 2).

**Table 2:** Risk of bias assessment of included studies using the newcastle–ottawa scale (NOS)

Study number	Study	Selection (max 4)	Comparaility (max 2)	Exposure/Outcome (max 3)	Total score
1	Sharpe, 1983	**4**	**1**	**2**	**7**
2	Goldenberg, 1986	**3**	**1**	**2**	**6**
3	Grossman, 1991	**3**	**1**	**2**	**6**
4	Leiguarda, 1997	**4**	**1**	**2**	**7**
5	Monza, 1998	**3**	**1**	**3**	**7**
6	Pharr, 2001	**4**	**1**	**3**	**8**
7	Peigneux, 2001	**4**	**2**	**3**	**9**
8	Soliveri, 2003	**4**	**2**	**3**	**9**
9	Soliveri, 2005	**4**	**2**	**3**	**9**
10	Soliveri, 2005	**4**	**2**	**3**	**9**
11	Boxer, 2006	**3**	**2**	**1**	**6**
12	Borroni, 2008	**3**	**1**	**2**	**6**
13	Gebhardt, 2008	**4**	**2**	**3**	**9**
14	Huey, 2009	**4**	**1**	**2**	**7**
15	Foki, 2010	**4**	**2**	**3**	**9**
16	Uluduz, 2010	**4**	**2**	**3**	**9**
17	Stamenova, 2011	**4**	**2**	**3**	**9**
18	Qureshi, 2011	**4**	**2**	**3**	**9**
19	Burrell, 2014	**4**	**2**	**3**	**9**
20	Kübel, 2017	**4**	**2**	**3**	**9**
21	Vanbellingen, 2018	**4**	**2**	**3**	**9**
22	Jo, 2021	**4**	**2**	**3**	**9**

### Praxis assessment method(s)

Below, we summarize the key methods used to assess different types of apraxic deficits across the included studies (Tables 1, 3, 4, 5 and 6).

**Table 3:** Deficits and diagnostic relevance of apraxia in PD

Study Number	Study	Assessment method(s)	Main apraxic deficit(s) in PD	Diagnostic/clinical significance
1	Sharpe, 1983	Gestural Test based on the work of Kaplan	Impaired imitation and pantomime of tool use; spatial errors on non-representational tasks	Motor planning impairment beyond basal symptoms
2	Goldenberg, 1986	Imitation of gestures; Pantomime of symbolic gestures and object use	Sequence imitation deficits	Correlated with visuospatial abilities
3	Grossman, 1991	Boston praxis battery	Impaired imitation of meaningless gestures and pantomime of transitive gestures	Occurs even in non-demented PD
4	Leiguarda, 1997	Imitation and pantomime of meaningful and meaningless gestures; MIT; Multiple step tasks	Transitive gesture impairment; spatial errors	Supports cortico-striatal dysfunction
5	Monza, 1998	De Renzi ideomotor apraxia test	Impaired sequential tapping and imitation of sequences of gestures	Rhythm production may affect sequencing
6	Gebhardt, 2008	CR & FT tasks	Impaired Dexterity	Suggests LKA rather than bradykinesia
7	Foki, 2010	CR task	Impaired Dexterity	Perirolandic dissociation with precentral overactivation and postcentral underactivation
8	Uluduz, 2010	The Mayo Clinic praxis test; Luria test; use of actual objects	Transitive/sequential impairment	IMA and IA in MSA and, to a lesser degree, in PD, independent of extrapyramidal signs
9	Qureshi, 2011	Sequencing picture tasks	Action-sequencing deficits	Supports IA in PD
10	Kübel, 2017	CR task	Impaired Dexterity	Dissociation from bradykinesia
11	Vanbellingen, 2018	CR task	Impaired Dexterity linked to QoL	Clinical relevance of LKA

*PD* Parkinson’s disease; *MIT* Movement imitation test; *CR* Coin rotation task; *FT* Finger tapping task; *QoL* Quality of life; *IMA* Ideomotor apraxia; *IA* Ideational apraxia; *LKA* Limb kinetic apraxia; *MSA* Multiple system atrophy

**Table 4:** Deficits and diagnostic relevance of apraxia in CBS

Study number	Study	Assessment method(s)	Main apraxic deficit(s) in CBS	Diagnostic/clinical significance
1	Pharr, 2001	TOLA	Impaired imitation and pantomime of transitive and intransitive gestures	More severe in CBS than PSP; aids differential diagnosis
2	Peigneux, 2001	Shortened version of the “Batterie d’Evaluation des Praxies”	Impaired imitation of meaningless gestures; imitation and pantomime of meaningful gestures; actual use of objects	Suggests distinct praxis networks
3	Soliveri, 2003	De Renzi ideomotor apraxia test	Distal gesture impairment	Differentiates CBS from PSP
4	Soliveri, 2005	De Renzi ideomotor apraxia test	Impaired imitation of intransitive gestures	Widespread gesture impairment
5	Boxer, 2006	Clinical examination	High prevalence of apraxia	Differentiates CBS vs. PSP
6	Borroni, 2008	De Renzi ideomotor apraxia test	Impaired imitation of intransitive gestures	Early tract abnormalities
7	Huey, 2009	TOLA	Impaired imitation of transitive and intransitive gestures	Linked to frontal/subcortical loss in CBS
8	Stamenova, 2011	Conceptual and production limb apraxia assessment	Impaired imitation and pantomime of transitive gestures	Both LHP and RHP patients were impaired in their ability to execute gestures (except in pantomime of intransitive gestures)
9	Burrell, 2014	Imitation of meaningful and meaningless hand gestures	Impaired imitation of meaningful and meaningless hand gestures	Apraxia correlates with pre-motor and parietal atrophy
10	Jo, 2021	Adaptation from the Florida Apraxia Battery	Impaired imitation of meaningless gestures; pantomime of transitive gestures	Distinct pathways of ideomotor and imitation apraxia in CBS

*TOLA* Test of oral and limb apraxia; *CBS* Corticobasal syndrome; *PSP* Progressive supranuclear palsy; *RHP* Right hemisphere predominance; *LHP* Left hemisphere predominance

**Table 5:** Deficits and diagnostic relevance of apraxia in PSP

Study number	Study	Assessment method(s)	Main apraxic deficit(s) in PSP	Diagnostic/clinical significance
1	Leiguarda, 1997	Imitation and pantomime of meaningful and meaningless gestures; MIT; Multiple step tasks	Transitive gesture impairment; spatial errors	Correlates with cognition
2	Monza, 1998	De Renzi ideomotor apraxia test	Sequential deficits	Cortical-subcortical involvement
3	Pharr, 2001	TOLA	Impaired imitation and pantomime of transitive and intransitive gestures	Milder than CBS
4	Soliveri, 2003	De Renzi ideomotor apraxia test	No clear distal–proximal dissociation	Distal impairment more CBS-like
5	Soliveri, 2005	De Renzi ideomotor apraxia test	Sequence errors	Suggests dysfunction of the anterior frontal cortical areas
6	Soliveri, 2005	De Renzi ideomotor apraxia test	Sequence errors	Emerges in advanced decline
7	Boxer, 2006	Clinical examination	Less frequent than CBS	Atrophy patterns aid differentiation

*TOLA* Test of oral and limb apraxia; *CBS* Corticobasal syndrome; *MIT* Movement imitation test

**Table 6:** Deficits and diagnostic relevance of apraxia in MSA

Study number	Study	Assessment method(s)	Main apraxic deficit(s) in MSA	Diagnostic/clinical significance
1	Leiguarda, 1997	Imitation and pantomime of meaningful and meaningless gestures; MIT; Multiple step tasks	No evidence of praxis impairment	Uncommon in early MSA
2	Monza, 1998	De Renzi ideomotor apraxia test	Sequential deficits	May reflect cognitive burden
3	Uluduz, 2010	The Mayo Clinic praxis test; Luria test; use of actual objects	Ideomotor and ideational errors	May occur in advanced MSA

*MSA* Multiple system atrophy; *MIT* Movement imitation test

Assessment of apraxia frequently relies on gestural tasks. Early work of Sharpe et al. [25] administered the Gestural Test, which is based on the framework of Kaplan, in order to assess gestural representation and spatial errors. The Gestural Test comprised two components examining the imitation and pantomime of implement usage and the imitation of meaningless gestures.

Building on this, Goldenberg et al. [26] examined a wide range of gestures, including the imitation of six finger postures, each involving two or three fingers; six positions of the hand; six movement sequences which required different combinations of simultaneous movements of arm, hand and fingers and a sequence of finger tapping which was demonstrated simultaneously to counting the number of tappings out loud and finally the pantomime of six symbolic gestures and six pantomimes of object use in order to examine IMA in PD patients compared to controls.

Similar methods of assessment were used across most studies of our review such as gestural tasks adopted from the praxis battery of the Boston Diagnostic Aphasia Examination [27] used by Grossman et al. [28], in order to asses apraxia in PD, Movement Imitation Test and Multiple Step Tasks alongside imitation and pantomime of gestures used by Leiguarda [29], examining IMA and IA in PD, PSP, and MSA, a shortened version of the “Batterie d’Evaluation des Praxies (2000) [30] used by Peigneux et al. [31] incorporating the assessment of actual tool use, examining also CA, in CBD patients compared to controls and the semi-structured approach followed by Burrell et al. [32] which also assessed apraxia through imitation and pantomime tasks in CBS patients.

A substantial number of the literature employed standardized, validated apraxia tools. The most widely used was the De Renzi Ideomotor Apraxia Test [33], which was applied in patients with PD, CBS, PSP, MSA and control subjects [34–38]. This 24-item scale includes 12 meaningful (e.g., military salute) and 12 non-meaningful gestures performed by both hands either with the whole limb (e.g., sign of the cross) or with the hand only (e.g., thumbs-up sign) [33]. As specified in the original normative data, a score below 53 (of a possible 72) indicates apraxia, a score above 62 is normal, and scores in between are borderline. Their team emphasized modality-specific assessment—comparing imitation (visual input) versus pantomime (verbal input) across transitive (object-related) and intransitive (symbolic) gestures. The neural overlap versus dissociation of these gesture types remains debated.

Other studies [39, 40] used Test of Oral and Limb Apraxia (TOLA) [41] which examines four types of gestures (with five gestures of each type): proximal intransitive (e.g., wave goodbye), proximal transitive (e.g., stir a gallon of paint), distal intransitive (e.g., make an okay sign), and distal transitive (e.g., turn a screwdriver) in order to examine apraxic errors in CBD and PSP. Qureshi et al. [42] administered sets of pictures that showed the steps in completing a task, but the steps were shown out of order to check for IA in PD while Uluduz et al. [43] examined IMA, IA and actual tool use using a broader praxis battery, the Mayo Clinic praxis test battery [44] and Luria Test. Both studies tested apraxia in PD compared to control subjects. More recent work of Stamenova et al. [45] examining CA and gesture production and Jo et al. [46] using an adaptation from the Florida Apraxia Battery evaluated extensively CA, IMA and IA in CBS.

Additional studies specifically targeted LKA using the Coin Rotation Task (CR) [47–50]. CR was administered in order to assess specifically LKA in patients with PD compared to ctrls. CR represents a measure of both finger dexterity and motor speed. In the CR task, the limb kinetic deficit is indicated by the reduced number of half turns per time unit and coin drops [51].

### Apraxia in PD

Of the 22 studies included in this review, 11 investigated apraxia in PD. Across these studies, the most consistent findings involved impairments reflecting IMA and LKA, with additional evidence for IA in some cohorts (Table 3).

#### PD versus healthy controls or/and without evidence of cerebral damage

Sharpe et al. [25] indicated that PD patients performed at a significantly lower gestural level on the representational tasks [F (1, 28) = 7.27, *p* < 0.05] and made significantly more spatial errors on non-representational tasks than the normal controls [F (1, 28) = 14.02, *p* < 0.001]. Their performance was better on imitation than verbal command [F, (1, 28) = 20.85, *p* < 0.00 l). According to the authors, the finding that PD patients show a deficit in gestural movements similar to patients with frontal lobe damage suggests either that basal ganglia-frontal lobe interactions are necessary for praxis or dysfunction of either one affects the total praxis function. Goldenberg et al. [26] reported IMA in a PD cohort compared to control subjects (*p* < 0.0003). Imitation of movement sequences (*p* < 0.0001) was affected more severely than performance of single movements. The degree of impairment was not related to severity of motor disability but correlated strongly with the results of tests that measured visuospatial and visuoperceptive abilities. Grossman and colleagues [28]confirmed Sharpe’s results [25] reporting that a praxis deficit may be evident in up to 64% of PD patients, indicating that apraxia may be a frequent and debilitating feature in PD.

Qureshi et al. [42] focused specifically on IA. Their study confirmed deficits in action-sequencing tasks in PD (*p* < 0.05), thus supporting the idea that IA, an action-sequence planning deficit, is a relevant feature of PD. They also supported that the errors were predominantly in sequencing rather than repetition or omission, indicating that the poor performance was not caused by perseveration.

Due to the controversy whether impaired fine motor control may stem from LKA rather than bradykinesia in PD, four studies in our review focused on assessing dexterity with the CR and bradykinesia with the FT in order to solve this issue. Gebhardt et al. [47] explored the effects of dopaminergic treatment on bradykinesia and dexterity. In patients with PD, regardless of hand involved, dopaminergic treatment only mildly improved CR performance, in contrast to the strong increase in FT scores (up to the level of controls), as demonstrated by the significant triple interaction of the factors group, medication, and task (F_1,22_ = 7.9, *p* = 0.01; η^2^ = 0.26). Furthermore, CR scores were considerably lower, both in “off” and “on” state, than in normal controls, pointing to a substantial impairment of dexterity in PD (*p* < 0.001). The authors concluded that impaired manual dexterity showing significantly diminished response to dopaminergic treatment suggests that dexterous deficits in PD are related to LKA rather than bradykinesia. In line with the results of Gebhardt et al. [47], Foki et al. [48] employed fMRI to examine specific brain activation patterns among PD patients with impaired performance in the CR task. PD patients' CR performance was worse as compared to controls (*t* = 6.349, *p* < 0.001). Further, CR performance and bradykinesia indices in the “off” state did not correlate significantly, which suggests an “apraxic” component as major contributor to impaired dexterity. According to the authors, this evidence and the fact that fMRI signals specific for bradykinesia should affect both the CR and the FT, render it likely that these results highlight brain network changes specific for the apraxia task.

Kübel et al. [49] further explored the hypothesis that dexterous deficits in PD are associated with altered activity and connectivity in left parieto-premotor praxis network. The study concluded that PD patients exhibit a neural correlate for the behavioral dissociation between LKA and bradykinesia. Finally, Vanbellingen et al. [50], also using the CR, pointed that the strong association of impaired dexterity (PD < ctrl; *p* < 0.0001) and quality of life is independent of bradykinesia, thereby underscoring the clinical relevance of LKA in PD.

#### PD versus other movement disorders and healthy controls

Three studies compared apraxia in PD versus other parkinsonian syndromes and control subjects. Initially, Leiguarda et al. [29] conducted a comparative study among PD, PSP and MSA and drug-induced parkinsonism (DIP). The study revealed that PD patients (almost ¼) exhibit bilateral IMA for transitive movements more frequently than those with MSA and DIP, but less so than PSP patients. In PD, transitive but not intransitive movements were affected, the patients made spatial errors in tasks when asked to imitate hand and finger postures, but none of them failed on pantomime comprehension and pantomime recognition/discrimination. Subsequently, Monza et al. [38] also compared IMA in PD, PSP and MSA using the De Renzi Ideomotor Apraxia Test [33]. Seven (50%) of 14 patients with PD scored between 53 and 62 (borderline) but none of the patients with PD scored below 53 (apraxia score). Performance imitating single and sequence of gestures differed significantly between groups (*p* = 0.002 and *p* < 0.0001, respectively), with all disease groups doing significantly worse than controls, and patients with PSP doing significantly worse than those with MSA or PD.

More recently, Uluduz et al. [43] found that mean total praxis score was significantly lower for patients with PD (92.4 ± 4) and MSA (75.9 ± 18) than for controls (97.4 ± 2) (p = 0.000). Transitive performances of upper extremities and sequential tasks were significantly impaired in patients with PD compared to control subjects (*p* < 0.05). Interestingly, there was no correlation between total praxis scores and sum scores of tremor, bradykinesia, and rigidity of both of the upper limbs of patients with PD, suggesting that apraxia in PD is independent of the classic extrapyramidal signs. It is important to mention that MMSE scores were within normal limits in the PD group, and there was no correlation between apraxia scores and MMSE, supporting pure apraxic deficits rather than global functioning ones leading to reduced performance on the praxis tests.

Overall, the evidence suggests that apraxia in PD most frequently manifests as ideomotor and limb-kinetic deficits, with additional evidence for ideational deficits related to action sequencing. Importantly, several studies indicate that impaired dexterity in PD cannot be fully explained by bradykinesia alone, supporting the presence of a praxis-related component.

### Apraxia in CBS

Apraxia is considered a hallmark of CBS. Ten studies in this review specifically investigated the clinical manifestations of apraxia in patients with CBS (Table 4).

#### CBS versus healthy controls

Six studies in our review compared CBS patients with control subjects. Although the primary focus of these studies was the examination of the neural underpinnings of apraxia in CBS, they provided important data regarding the clinical manifestations of apraxia. Peigneux et al. [31] using PET imaging for the neural correlates and a shortened version of the “Batterie d’Evaluation des Praxies” [30] revealed that global mean performance score and global mean correction score were lower in the CBD population than in the control group (*p* < 0.001). Pantomime to object and actual use of objects conditions could not be performed in four cases out of eighteen. In Borroni’s et al. study [37], most patients, despite an asymmetrical onset, showed bilateral apraxia: only 3 patients presented with right unilateral apraxia. Mean (SD) test scores on the test of De Renzi et al. [33] were 51.0 (23.8) for the right limb and 52.6 (21.9) for the left limb. A year later, Huey et al. [40], trying to determine the brain areas associated with specific components of IMA in CBS, administered TOLA [41] in 48 CBS patients. In their study, patients with CBS performed significantly worse than the healthy controls on the TOLA measures (TOLA imitation: *t* = 13.6, *p* = 0.01; command: *t* = 11.4, *p* = 0.01; oral: *t* = 6.1, *p* = 0.01; limb: *t* = 13.6, *p* = 0.01). The overall degree of apraxia was independent of the side of motor impairment. Praxis to imitation (vs command) was particularly impaired in the patients with CBS. Patients demonstrated equal impairment in transitive and intransitive gestures.

Stamenova and colleagues [45] evaluated the severity of apraxic deficits, grouped by the initially affected hemisphere: ten with right hemisphere predominance (RHP) and seven with left hemisphere predominance (LHP). Both groups showed preserved conceptual knowledge of tools and actions but were impaired on gesture production compared to controls. Transitive gestures were more affected than intransitive, and imitation was more impaired than pantomime, indicating visuomotor deficits. Verbal cuing worsened imitation performance in LHP patients. Burrell et al. [32] showed that patients with CBS had moderate motor functional disability, due predominantly to limb apraxia, parkinsonism, and rigidity, rather than weakness. All patients in their study had difficulty producing meaningful and meaningless hand gestures. There was no significant difference between the mean limb-meaningful and limb-meaningless scores in CBS patients. Finally, Jo et al. [46] reported that among 52 CBS patients, the most common apraxia subtype was the presence of both ideomotor (the term is used to describe deficits in pantomimes according to the authors) and imitation apraxia (the term is used to describe deficits in imitation of meaningless gestures according to the authors) (*n* = 22, 42.3%), followed by ideomotor apraxia only (*n* = 15, 28.8%) and imitation apraxia only (*n* = 15, 28.8%). Patients exhibiting both subtypes were more likely to display typical CBS and progressive supranuclear palsy features.

#### CBS versus PSP and healthy controls

Four studies compared apraxia in CBS, PSP and control subjects. Pharr et al. [39] found that both CBS and PSP patients made more apraxic errors on both transitive and intransitive tasks than controls (*p* < 0.001), but severity was greater in CBS (*p* < 0.001). Soliveri et al. [34] further demonstrated that CBS patients showed significantly greater deficits in distal than proximal movements (*p* = 0.007), consistent with LKA. Two years later, their team reported that despite similar cognitive impairment, apraxia was more frequent in CBS (70.8%) than PSP (36%), but only when assessing the most compromised hand (*p* < 0.001) [36]. They also showed that CBS patients present more errors of awkwardness and simple gesture impairment, whereas PSP patients make more sequence errors. Boxer et al. [52] corroborated these findings, showing that apraxia was significantly more prevalent in CBS than in PSP (*p* < 0.001).

Overall, these studies show that apraxia in CBS is common. Multiple components of praxis -including ideomotor, ideational, and limb-kinetic deficits- may coexist, reflecting widespread disruption of frontoparietal praxis networks. Compared with other parkinsonian syndromes, particularly PSP, patients with CBS consistently demonstrate more severe and distinctive apraxic impairment patterns.

### Apraxia in PSP

Building on the comparative findings with CBS, the studies included in this systematic review indicate that apraxia also occurs in PSP, though typically with lower prevalence and milder severity. Whereas CBS is marked by frequent severe LKA, IMA and IA, PSP tends to exhibit more frequently sequence errors (IA) and milder apraxic deficits on both transitive and intransitive gestures (IMA) but LKA is not dominant. The following studies specifically evaluated the prevalence, diagnostic significance and clinical manifestations of apraxia in PSP, allowing further delineation of its distinct phenotype within the spectrum of APS (Table 5).

#### PSP versus other movement disorders and healthy controls

Leiguarda et al. [29] examined 12 PSP patients and compared them with PD, MSA, DIP and control subjects. They found bilateral IMA for transitive (75%) and intransitive (25%) movements in PSP patients (*p* < 0.006). Errors were predominantly spatial, including body-part as object, trajectory and external/internal configuration errors. In line with PD patients mentioned above, none of the PSP patients failed on pantomime comprehension and pantomime recognition/discrimination. Some PSP patients exhibited, in addition, LKA and a minority of them displayed deficits on tasks involving multiple steps (possibly reflecting the presence of IA). IMA scores correlated significantly with global cognitive impairment as measured by MMSE.

Monza et al. [38] reported the most severe deficits in sequential tapping and the imitation of sequences of gestures in a PSP cohort (*p* < 0.0001) among PSP, PD and MSA groups. However, based on IMA scores and a qualitative analysis of errors, only three patients with PSP were considered apraxic. It is important to mention that the impairment of patients with PSP was more pervasive than that of the other groups, and included compromise of visuospatial functions, attention, and memory.

Soliveri et al. [35] studied 25 probable PSP patients, including 11 demented (D-PSP) and 14 non-demented (ND-PSP) individuals, compared with 19 healthy controls. PSP patients exhibited sequence errors and poorly executed complex gestures, with more pronounced deficits in D-PSP but also compromised total and partial apraxia scores in the ND-PSP group. ND-PSP cohort showed prominent difficulty in non-significant rather than significant gestures (*p* = 0.003). The authors conclude that this finding supports the interpretation of a frontal origin of limb apraxia in PSP.

In general, apraxia in PSP is less prevalent and milder than in CBS, mostly disrupting gesture sequences and complicated actions and associated with cognitive deterioration. In these particular studies, cognitive dysfunction, particularly frontal-executive impairment, appears to modulate apraxia severity in PSP. However, impaired cognitive and praxis profiles vary among PSP phenotypes.

### Apraxia in MSA

Across the three studies that assessed praxis in MSA, findings were heterogeneous and inconsistent, with the earlier study reporting preserved praxis functions whereas later studies demonstrated distinct apraxic manifestations (Table 6).

#### MSA versus other movement disorders and healthy controls

The earlier study by Leiguarda et al. [29], in which an extensive protocol of apraxia assessment (as mentioned above) was administered to 10 patients with MSA, did not report apraxic deficits. More specifically, none of the MSA patients showed signs of any praxis disturbance and all the patients performed normally on the MIT (Mean = 34.1 ± 1.2, cut-off: 33) and Multiple Step Tasks while mean ideomotor apraxia scores for transitive (Mean: 29.6 ± 0.6, cut-off score: 26) and intransitive (Mean: 19.8 ± 0.6, cut-off score: 19) movements didn’t fall below the cut-off point for ideomotor apraxia. While bilateral IMA for transitive movement was identified in 2/3 of PSP patients and almost 1/3 of PD patients, patients with MSA performed comparably to controls on all praxis measures.

On the other hand, Monza et al. [38] reported mild deficits in apraxia. According to De Renzi et al. [33] scoring, IMA is certainly present when a total score is less than 53 and certainly absent when a total score is greater than 62, with a score between 53 and 62 considered borderline. Four (20%) of 19 patients with MSA scored between 53 and 62 (borderline) and 2 (10%) of 19 patients with MSA scored less than 53. Qualitative analysis of uncorrected gestures showed that patients with MSA often made more than 1 type of error (similarly with PSP patients as it is discussed above): 85% of the errors made by patients with MSA were due to clumsiness, and 15% were due to sequence errors. However, it is important to mention that in this MSA cohort, patients were compromised compared with control subjects in all cognitive functions.

Finally, Uluduz et al. [43] recruited an extensive assessment of apraxia which comprised the Mayo Clinic praxis test, Luria test and the use of actual objects. Their findings reveal reduced total praxis scores in MSA patients (75.9 ± 18) compared with both PD (92.4 ± 4) patients and control subjects (97.4 ± 2) (*p* = 0.000). In patients with MSA, all subgroup items of the praxis scores were significantly worse than those of the control subjects and exhibited more errors, while performing the ideomotor praxis test than patients with PD or the control group. Errors were observed in the use of a body part as an object and/or in trajectorial movements, such as hypersupination of the forearm or elevation of the arm above the head during finger tapping, and abduction and adduction of the whole arm during alternating movements of the hands, even though the required movements were demonstrated to them. The authors conclude that ideomotor and ideational praxis dysfunctions were prominent in the MSA group. However, patients with MSA displayed more cognitive impairment, anxiety, and depression compared to controls which may correlate with abnormalities in performing praxis tests.

Overall, evidence suggests that apraxia can occur in MSA, however it may only affect a small percentage of patients, it seems less common and less severe than in PSP and CBD and it is linked to cognitive dysfunction. Only a small number of studies have examined apraxia in MSA and their findings are contradictory. In addition, differences in assessment methods further limit comparability. Hence, no reliable conclusions can yet be drawn regarding the prevalence and manifestations of apraxia in MSA.

## Discussion

To our knowledge, this is the first systematic review that synthesized the evidence on limb apraxia in PD and APS, with the aim of delineating disease-specific praxis profiles. Across the included studies, apraxic deficits emerged across PD, CBS, PSP and MSA, but with differences in frequency, severity and qualitative error patterns. Taken together, the findings support the view that praxis dysfunction in parkinsonian disorders reflects a disturbance of higher-order action control that can contribute to disability beyond classic extrapyramidal motor symptoms.

Apraxia in PD has often been described as mild and more prominent in later disease stages (Hoehn and Yahr stage 3 and higher). However, several strands of evidence, both within the included studies and in broader literature, support a clinically meaningful impairment in dexterous, skilled movements that may not be fully captured by bradykinesia or rigidity alone [53, 54]. Impaired finger dexterity leading to difficulties such as using computer keyboards, fastening buttons or tying laces is commonly reported by PD patients. There is a controversy in literature regarding the possibility of an apraxic, that is, a cognitive-motor nature of dexterous deficits in PD rather than motor deficits such as bradykinesia [47, 51, 55]. Vanbellingen et al. [54] tried to explore the relationship of finger dexterity as measured by CR with ideomotor praxis function assessed by the test of upper limb apraxia (TULIA) [56] and parkinsonian symptoms using the revised version of Movement Disorder Society-Unified Parkinson’s Disease Rating Scale (MDS-UPDRS) [57] in non-demented patients with PD. Their study showed that CR significantly correlated with TULIA irrespective of the motor state and arm involved, but not with the MDS-UPDRS. This association was significantly influenced by Hoehn and Yahr stage. The authors conclude that the strong association of finger dexterity with praxis function but not the parkinsonian symptoms indicates that impaired finger dexterity in PD may be indeed apraxic in nature. Furthermore, this association is influenced by disease stage, being more pronounced in advanced PD when cortical pathology is expected to develop.

The results of the present systematic review are consistent with these findings, showing that impaired finger dexterity cannot be fully explained by bradykinesia, rigidity or tremor, highlighting the cognitive-motor interface of praxis dysfunction in PD. On the other hand, while the literature portrays apraxia in PD as late-onset, mild and primarily manifesting as LKA affecting finger dexterity, the studies included in our review support that it may emerge earlier and IMA may be present as well. One contributing factor is the lack of standardized assessment tools and protocols tailored to these conditions, which hinders accurate evaluation and comparison of apraxic deficits. This underscores the need to assess apraxia systematically in larger samples with validated tools to draw reliable conclusions. These should include standardized batteries, multimodal evaluation approaches (e.g., kinematic analysis, neuroimaging), and clearly defined diagnostic criteria to ensure greater diagnostic specificity and reproducibility.

Limb apraxia is a core clinical feature of CBS [16], often appearing early in the disease course and more severely than in PD, PSP and MSA [18]. Impairment in finger dexterity, imitation and pantomime of gestures, action-sequencing and actual tool use can all be present simultaneously reflecting the most severe and widespread apraxic deficits among the disorders examined. A key qualitative marker in CBS is disproportionate impairment in imitation of meaningless gestures, which is consistent with disruption of dorsal visuomotor transformation pathways. This aligns with recent morphometric work localizing apraxia severity and specifically linking deficits in meaningless gesture imitation with superior parietal and inferior parietal (angular/supramarginal) involvement [58]. Such findings strengthen the neurocognitive coherence of the CBS apraxia phenotype: the more the parietal-premotor praxis circuitry is compromised, the more pronounced the imitation deficits tend to be. In addition, patients present core deficits in action knowledge and mechanical problem solving, which has also been linked to parietal lobe pathology. General semantic memory may also be affected in CBS in some cases and this may then contribute to impaired object usage [59].

An additional point that has practical diagnostic implications is that CBS is a clinical syndrome with heterogeneous underlying pathologies. CBS can arise from CBD pathology but also from AD and other underlying pathologies, and this neuropathologic diversity may contribute to variability in cognitive-praxis profiles across cohorts [60]. From a clinical standpoint, this underscores the value of pairing praxis phenotyping with biomarkers when available, since a “CBS phenotype” does not guarantee a single pathological substrate.

From a measurement standpoint, the literature increasingly supports structured apraxia instruments as clinically useful. Work applying the Goldenberg Apraxia Test (GAT) has shown value in quantifying apraxia and localizing its neural correlates in CBS [61]. Goldenberg’s studies [62–65] have supported that imitation of hand postures relies on the topographical knowledge about body parts because the patient cannot see the final posture that they are carrying out, indicating a major involvement of the left parietal lobe. On the other hand, visuo-spatial abilities play an important role in the imitation of finger postures, such that the right hemisphere, in addition to the left, is also responsible for the task. Regarding pantomime production, pantomime of tool use places exceptionally high demands on selection and combination of distinctive features of objects and actions and remains hard to interpret due to the distinct cognitive processes it involves [66]. According to his observations, an extensive assessment of apraxia should comprise the examination of: a) imitation of meaningless and meaningful gestures b) mechanical problems for assessing practical knowledge c) picture-matching tasks for assessing conceptual knowledge d) pantomime e) actual tool use in isolation f) actual tool use with corresponding objects g) novel tools. Beyond localization, such standardized tools may improve differential diagnosis and allow more sensitive tracking of progression and treatment response than informal bedside assessment.

Apraxia in PSP is less extensively studied and often appears milder than in CBS, but the available evidence supports a pattern that is frequently weighted toward sequence errors and difficulty with complex actions, especially in the context of cognitive decline. A key study [35] reported that apraxia in PSP correlated strongly with dementia and that patients primarily committed sequence errors and executed complex gestures poorly, supporting the role of frontal dysfunction in the expression of limb apraxia in PSP.

Given the recognized phenotypic heterogeneity of PSP, praxis assessment could be particularly informative for identifying subgroups with greater cortical involvement and for improving both clinical characterization at age onset and monitoring of disease progression. In addition, since PSP can present with prominent speech/language impairment in some phenotypes, future work could examine how praxis deficits co-vary with broader frontostriatal cognitive profiles.

Finally, evidence for apraxia in MSA remains limited and mixed, with some cohorts showing preserved praxis and others demonstrating ideomotor/ideational disturbances. One plausible interpretation is that praxis impairment in MSA may emerge more clearly in subsets of patients with greater cognitive burden, but current data are insufficient to draw firm conclusions. Methodological factors likely contribute substantially: different studies used different batteries, cut-offs and operational definitions of apraxia, limiting comparability. In general, cognitive decline may occur in early stages but is generally common in advanced disease [67] and is often correlated with disease duration [68]. These findings are also consistent with our results, which show that apraxia can occur in MSA but is most commonly linked to cognitive deterioration.

A recurring limitation across the field is heterogeneity in apraxia assessment. Many studies rely on gestural imitation/pantomime tasks, but fewer adopt validated batteries across disease groups. Moving forward, a pragmatic approach for clinical and research settings could involve: a) a brief standardized screening tool for gesture production (meaningful + meaningless, imitation + pantomime); b) a dexterity measure sensitive to LKA (e.g., CR); c) an adjunct conceptual/action knowledge component and d) an action-sequencing measure.

Such approaches would improve comparability across studies and help disentangle deficits driven by motor impairment, executive dysfunction, visuospatial constraints and core praxis disruption.

Another gap in the literature is the lack of studies assessing apraxia across all major neurodegenerative parkinsonian disorders in a standardized manner. Due to the aforementioned heterogeneity in clinical assessment of apraxia among studies, safe conclusions regarding possible differences in apraxia frequency, severity and type of errors among parkinsonian disorders would require a large-cohort study with adequate representation of PD, PSP, MSA and CBS patients.

Across parkinsonian disorders, apraxia can substantially impair activities of daily living (e.g., dressing, utensil use, handling keys/phones) and may compound motor deficits. The PD literature provides especially clear evidence linking LKA/dexterity impairment to daily function and quality of life [69]. From a management perspective, modern apraxia literature emphasizes that recognition of praxis impairment is clinically meaningful because it may guide targeted rehabilitation and compensatory strategies (e.g., task-specific training, cueing approaches tailored to the impaired route) and it may also affect safety and caregiver burden. While robust interventional evidence in parkinsonian disorders is still emerging, systematic identification of apraxia is a prerequisite for treatment development and for selecting appropriate functional outcomes in trials [70]. Early diagnosis and appropriate management of apraxia are therefore essential for optimizing diagnostic accuracy, informing care strategies and improving rehabilitation outcomes in patients with neurodegenerative movement disorders.

### Limitations

This review has several limitations. First, the number of available studies remains relatively small, particularly for PSP and MSA. Second, substantial heterogeneity in assessment tools and outcome measures limited direct comparability across studies and precluded meta-analysis. Although the methodological quality of the included studies was generally moderate to high according to the NOS, several studies included relatively small cohorts and limited adjustment for potential confounding variables. An additional limitation is potential publication bias, as negative findings may remain unpublished.

### Implications for future research

Future research should employ standardized and multimodal praxis assessment protocols in larger, biomarker-confirmed cohorts, while systematically accounting for disease subtypes and clinical heterogeneity. Studies should include detailed phenotyping across the full spectrum of APS variants (e.g., PSP-Richardson, PSP-CBS, frontal or speech/language presentations) and investigate the functional consequences of apraxia through validated activities of daily living measures. Longitudinal studies are also required to clarify how praxis deficits evolve over time and whether they may serve as markers of cortical involvement or progression.

## Conclusions

This systematic review confirms that limb apraxia represents a clinically and diagnostically meaningful disorder across diverse neurodegenerative movement disorders, with distinct phenotypic profiles in PD and APS. CBS presents with the most severe and pervasive praxis impairment, affecting imitation and pantomime of meaningless and meaningful, both transitive and intransitive, gestures, as well as action sequencing, in addition to fine motor coordination. Traditionally apraxia in PD is considered to present predominantly as late-onset, mild LKA. However, our review supports that PD patients may exhibit a variety of apraxic deficits, resembling CBS. Importantly studies directly comparing CBS with PD are lacking. PSP patients exhibit milder, mainly sequence-related impairments and MSA presents heterogeneous, less pronounced praxis disturbances. Overall, systematic and standardized praxis assessment may improve differential diagnosis, functional profiling and the development of targeted rehabilitation strategies.

## Data Availability

No new data was produced for the purposes of this study.
